# Supplementary material for: Evaluating InferVision’s Computer-Aided Detection (CAD) algorithm for Tuberculosis (TB) screening, Lusaka, Zambia
Source: PLOS Glob Public Health. 2025 Jun 18;5(6):e0003955. doi: 10.1371/journal.pgph.0003955 (PMC12176154; doi:10.1371/journal.pgph.0003955)

The following shows a chi-squared test of association analysis conducted to determine whether observations with a score suggestive of TB were more or less likely to have missing data for the age variable than observations with a score not suggestive of TB.

If observations with missing data (which may be excluded from the analyses) appear to be similar to those without missing data, we can be reassured that their exclusion is unlikely to bias our conclusions. However, if observations with missing data are more or less likely to experience the outcome of interest (and are excluded from the analysis on which our final conclusions are based), this would have biased our conclusions. Therefore, we would have noted this as a potential limitation in our discussion.

The chi-squared test results below indicate that 270 (21.88%) observations with an “abnormal cad score,” and 116 (17.68%) observations with a “normal cad score” had missing data. These percentages are **not similar** (**chi-squared test p-value = 0.031**), indicating that the extent of missing data for the confounders of interest is **significantly associated with the outcome**. Excluding the observations with missing age would have biased our conclusions; hence the reason why we did not exclude these observations from our analysis.


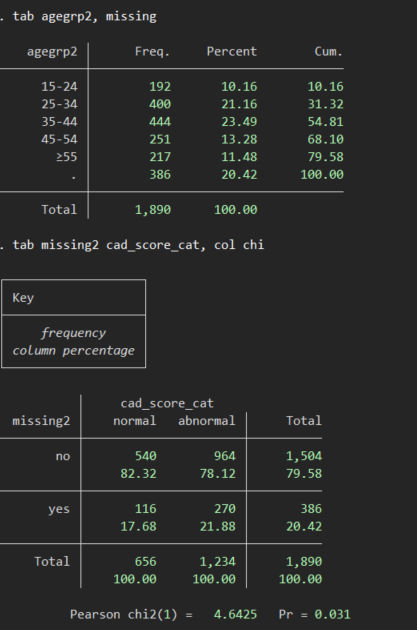


In response to the request to demonstrate how the sensitivity analysis was conducted, the image provided below illustrates the use of the user-defined **diagt** command in Stata, which is commonly used for diagnostic test evaluation. In this analysis, the variable **tb_final** serves as the composite (Xpert MTB/RIF and Culture test results) reference standard variable, where a value of 0 indicates a confirmed TB-negative case and a value of 1 indicates a confirmed TB-positive case. The variable **cad_score_cat** represents the binary classification of CAD scores, with **0 assigned to all TB scores less than 0.12** and **1 assigned to scores equal to or greater than 0.12**. This setup allowed us to calculate the sensitivity, specificity, and other diagnostic accuracy measures based on the TB scores observed.


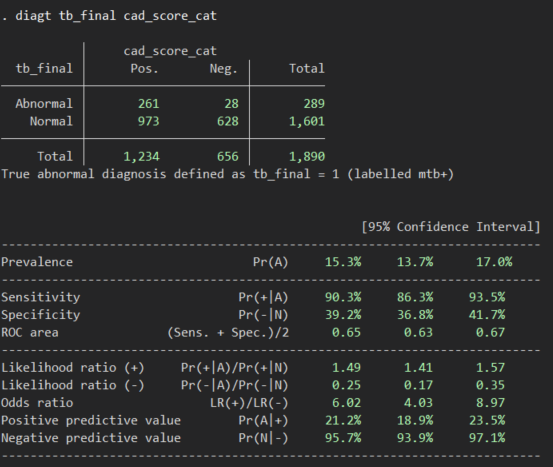

Supplement: S1 File — (ZIP) [file pgph.0003955.s001.zip › Supplemenatary file.docx]
