# Supplementary material for: Evaluating InferVision’s Computer-Aided Detection (CAD) algorithm for Tuberculosis (TB) screening, Lusaka, Zambia
Source: PLOS Glob Public Health. 2025 Jun 18;5(6):e0003955. doi: 10.1371/journal.pgph.0003955 (PMC12176154; doi:10.1371/journal.pgph.0003955)
Supplement: Attachment — Submitted filename: PGPH-D-24-01483_reviewer_Report.pdf [file pgph.0003955.s002.pdf]

Manuscript ID: PGPH-D-24-01483

Manuscript Title: Evaluating the Performance of InferVision's Computer-Aided Detection (CAD) Algorithm for Tuberculosis (TB) Screening

Authors: Paul Somwe, Minyoi Maimbolwa, Kanema Chiyenu, Mary Kagujje, Monde Muyoyeta

General comments

Thanks for granting me the opportunity to review this interest material. Notwithstanding the advancement in the development of diagnostic tools for TB, there still remains a big number of missed cases that may be attributable to inability to access care due to a number of reasons including costly diagnosis or poor infrastructure hindering access to diagnosis. Poor communities in the hard to reach areas of resource limited settings bears the greatest burden of the missed TB cases. Availability of tools such computer detection that can be adapted for point of care screening/diagnosis of TB may help bridge the gap. Hence, evaluation of such tools to establish their suitability is paramount. The authors in this article have retrospectively analyzed the performance of InferRead DR chest used to score digital chest images of approximately 2000 individuals aged 15+ years for diagnosis of TB. The findings suggest that for the derived threshold score of 10, InferRead DR chest achieved a high sensitivity $> 90\%$ but with a lower specificity. Thus, it may meet the WHO minimum Target Product Profiles for use a triage test to rule out TB. The findings adds to the rich literature that have demonstrated the value of artificial intelligence-based tools used to analyze digital chest images for the diagnosis of TB. Hence, important for the public to know. However, I have a few comments that may help improve the value of the manuscript if considered. This are detailed below. First, I present the general comments then for each specific part of the manuscript.

- How did you handle participants with Xpert trace results
- The authors refers Xpert as the "gold standard". This gives an impression that Xpert diagnoses TB with certainty. This is not true as Xpert is know to have false positive and false negative rates. Using the term reference standard may help acknowledge the limitations of Xpert. These limitations may need to be highlighted in the discussion as potential limitations to accurate evaluation of InferRead DR Chest. How did the InferRead DR chest threshold vary in the different sub-populations mentioned and how does these thresholds impact clinical decisions? Do you think this screening test can be applied elsewhere using the same threshold? Do you think evaluation in different regions/sub-populations may need to be conducted to understand the performance of the tool before it can be used?
- How do the findings established for InferRead DR chest compare to those of Computer-Aided Detection for TB version 6? Version 7?
- Do you think there is a possibility of bias introduced by the participants with missing Xpert results? Did you evaluate the distribution of their InferRead DR chest scores compared to those who had Xpert results?
- You stratified the analysis by age. Did you impute the missing age for the participants who had missing age to mitigate the potential bias? If not, this may need to be highlighted as the limitation in the study.

Section Specific comments

Title

As the performance of the CAD software varies from region to region, it would be helpful to mention the region/country where the study was conducted.

Abstract

Methods

- Age, HIV status and history of previous TB are mentioned as the stratifying variables but no definition of the population whose images were analyzed. Which population was considered? What was the inclusion criteria?
- Where was the study conducted? i.e From which region/country were the data collected? This should be included in the methods section.
- Lines 39 & 41: The authors refers Xpert as the "gold standard". This gives an impression that Xpert diagnoses TB with certainty. This is not true as Xpert is know to have false positive and false negative rates. Using the term reference standard may help acknowledge the limitations of Xpert. These limitations may need to be highlighted in the discussion as potential limitations to accurate evaluation of InferRead DR Chest.
- Line 44: Use of the term "Previous history of TB". I think "history of previous TB" sounds more appropriate.

Results

- In the methods section, estimation of the performance of InferRead DR Chest as a function of age, HIV status and history of previous TB is reported to have been conducted. However, there are no results. Can these be reported?
- Adding descriptive results of the population might help to quickly understand the sample analyzed upon reading the abstract.

Conclusion

- The authors make a conclusion on the need for further training of InferRead DR chest. This is not supported by the data in the abstract. I would recommend adding results to justify this claim.

Manuscript

Study Design & Procedures

- Line 106-107: The authors wrote that a score closer to or equal to 100 indicate the likelihood of TB-related abnormality. While higher scores are associated with higher likelihood of TB, this sentence is not correct as the authors established a threshold score of 10 as suggestive of TB. I suggest this sentence be rewritten.

Data Analysis

- Line 112: The use of the term "gold standard" needs attention as mentioned in the general comments and in the abstract above. Even culture, which is known to have a higher accuracy for TB diagnosis has less than optimal sensitivity. Hence, it can't be referred to as a gold standard as reported by the authors in line 263.

- Lines 115-117: As one of the goals of the study was to determine a feasible threshold score for evaluation of InferRead DR chest, shouldn't this be presented in the results section?

- Line 127: InferRead Chest DR? or InferRead DR Chest?

Conclusions

As mentioned in the general comments above, this section may need to be expanded. For example, give a comparison of InferRead DR Chest to other AI-based methods such CAD4TBv6 & V7. For my own understanding, the threshold score of 10 derived for this screening tool is close to the values of 10 & 18 for CAD4TB version 7 obtained elsewhere (`https://doi.org/10.1371/journal.pone.0305126`). Are they based on the same AI algorithm? Handling of Xpert trace results may need to be mentioned. You mentioned the need for further training of InferRead DR chest to improve accuracy. Given the performance and the possibility for improved accuracy upon further advancement, is this screening tool affordable? Does it meet the other WHO recommended properties for use as a triage test?
