# Supplementary material for: Evaluating InferVision’s Computer-Aided Detection (CAD) algorithm for Tuberculosis (TB) screening, Lusaka, Zambia
Source: PLOS Glob Public Health. 2025 Jun 18;5(6):e0003955. doi: 10.1371/journal.pgph.0003955 (PMC12176154; doi:10.1371/journal.pgph.0003955)
Supplement: Attachment — Submitted filename: PGPH-D-24-01483_R1_Report_01252025.pdf [file pgph.0003955.s004.pdf]

January 25, 2025

The Academic Editor
PLOS Global Public Health

Dear Emily,

RE: Manuscript Review

Thank you for giving me the opportunity to review the manuscript entitled: ***Evaluating InferVision's Computer-Aided Detection (CAD) Algorithm for Tuberculosis (TB) Screening, Lusaka Zambia***, **Manuscript ID: PGPH-D-24-01483R1**. The authors responded to my concerns very well, and I am satisfied. I found the manuscript to be very interesting and flowing now. Nonetheless, I still have a few comments that may help improve the clarity of the manuscript if addressed. My apologies for the delay occassioned by the technical hitches with my email account.

Yours sincerely,

Alfred Keter

Manuscript ID: PGPH-D-24-01483R1

Manuscript Title: Evaluating InferVision's Computer-Aided Detection (CAD) Algorithm for Tuberculosis (TB) Screening, Lusaka Zambia

Authors: Paul Somwe, Minyoi Maimbolwa, Kanema Chiyenu, Mary Kagujje, Monde Muyoyeta

Abstract

- Lines 38 & 56: The reported age need to include units (years)
- Line 41: The authors specified the "WHO target product profile" as "WHO target profile". This should be specified correctly.
- Line 61: "tb" should be capitalized i.e. "TB" as it is an acronym

Introduction

- Lines 94 & 95: References 19 & 21-22 are disjoint
- Line 102: The cited reference for the WHO TPPs is not correct; Consider the reference in this link `https://iris.who.int/bitstream/handle/10665/135617/WHO_HTM_TB_2014.18_eng.pdf`

Methods

Study Design

No comments.

Participants

- Line 142: Might be helpful to add the facility and community-based approaches?

Test Methods

- Line 149-150; The statement " ... a threshold score of $\geq$0.1 was established as suggestive of TB in this study." is a finding and should be reserved to the **Results** section.
- Lines 152-154: Was composite reference standard derived? If so, it needs to be spelt out clearly. See Table 1.

- Line 155: This sentence mentions the findings "... was a score of ≥0.1". Delete this to make the senetecnce grammaticallly correct. Findings should go to the **Results** section.

- Line 155-158: Consider revising the reference to the correct source in `https://iris.who.int/bitstream/handle/10665/135617/WHO_HTM_TB_2014.18_eng.pdf`

Data Analysis

- Line 163-164: The statement " ... with a score of ≥0.1 identified as meeting the WHO recommended sensitivity of 90%" is a finding and should be reserved to the **Results** section.

- Line 166: How was the reference standard defined? If a composite of Xpert MTB/RIF and Culture then that should be mentioned explicitely in this subsection. Table 1 seems to suggest that a composite reference standard was derived.

- Lines 175-177: The justification for the calculating the NNS should be reserved to the discussion. Please move it.

Results

- Line 240-241: This sentence seems to suggest that the threshold was set to allow evaluaion of the index test. This is not true as the threshold was determined based on the WHO recommended minimum requirements on the sensitivity of the targeted product. Add a sentence before this sentence to reflect it as such. The sentence included in the abstract is well written.

- Line 254: "Based on the set threshold of $\geq$ 10," This threshold was not set but determined based on WHO minimum requirement of 90% sensitivity set by WHO for targeted products.

- Lines 254-256: Revise this sentence as mentioned in the second bullet above.

- Lines 267: "... and individuals with history of previous TB showed" needs to be revised to read "... and individuals with a previous TB history of previous TB yielded"

- Table 1 & Table 2: Revise ”Previous history of TB” to ”History of previous TB”
- Table 1: HIV status results - ”Indeterminant” or ”Indeterminate”. I think the later.

Discussion

- Lines 313-314: This interpretation is incorrect and even contracdicts the interpretation in Lines 337-340. The specificity of InferRead DR Chest were similar for males both females. While the female gender had a higher specificity, the 95% confidence intervals of specificity for both males & females are overlapping. Hence, the difference is not statistically significant.
