# Supplementary material for: Evaluating InferVision’s Computer-Aided Detection (CAD) algorithm for Tuberculosis (TB) screening, Lusaka, Zambia
Source: PLOS Glob Public Health. 2025 Jun 18;5(6):e0003955. doi: 10.1371/journal.pgph.0003955 (PMC12176154; doi:10.1371/journal.pgph.0003955)
Supplement: Attachment — Submitted filename: Response to Reviewers.docx [file pgph.0003955.s005.docx]

**20^th^ September 2024**

**Emily B. Wong**

Academic Editor

PLOS Global Public Health

Dear Emily,

We would like to thank you and the reviewers for their valuable comments and feedback on our manuscript titled “**Evaluating the Performance of InferVision’s Computer-Aided Detection (CAD) Algorithm for Tuberculosis (TB) Screening**”. We appreciate the thorough review process and are grateful for the opportunity to improve our work. Below, we have addressed each of the reviewers' comments in detail. Our revisions are highlighted as ***tracked changes*** in the manuscript to reflect the changes based on these comments.

**Reviewer #1 Comments:**

**Comment 1:** How did you handle participants with Xpert trace results?

**Response:** In our study, we made the decision to exclude Xpert trace results from the reference standard. This choice was based on the understanding that trace results indicate an indeterminate or borderline positive status, which does not meet the criteria for a definitive TB diagnosis.

**Comment 2:** The authors refers Xpert as the “gold standard”. This gives the impression that Xpert diagnoses TB with certainty. This is not true as Xpert is known to have false positive and false negative rates. Using the term reference standard may help acknowledge the limitations of Xpert. These limitations may need to be highlighted in the discussion as potential limitations to accurate evaluation of InferRead DR Chest. How did the InferRead DR chest threshold vary in the different sub-populations mentioned and how does these thresholds impact clinical decisions? Do you think this screening test can be applied elsewhere using the same threshold? Do you think evaluation in different regions/sub-populations may need to be conducted to understand the performance of the tool before it can be used?

**Response:** We appreciate your suggestion to use the term “reference standard” to better reflect the inherent limitations of the Xpert test, which can indeed yield false positives and false negatives. We have revised the manuscript to refer to “reference standard” to ensure clarity and to acknowledge the limitations of Xpert. Additionally, we have highlighted this limitation in the discussion section as a potential factor that may influence the accurate evaluation of the InferRead DR Chest tool.

Regarding the variation of the InferRead DR Chest threshold in different sub-populations, we observed differences in performance metrics across groups defined by age, sex, history of previous TB and HIV status. These variations are significant because they could inform clinical decision-making, particularly in tailoring screening strategies for specific populations. We recognize that while the threshold of ≥0.1 was selected based on its alignment with WHO guidelines, the applicability of this threshold in different regions or sub-populations may require further validation studies.

**Comment 3:** How do the findings established for InferRead DR chest compare to those of Computer-Aided Detection for TB version 6? Version 7?

**Response:** A cited study in our manuscript indicated that CAD 4 TB and qXR achieved an AUC of ≥85 on the same dataset, which reflects a much better performance compared to our findings.

**Comment 4:** Do you think there is a possibility of bias introduced by the participants with missing Xpert results? Did you evaluate the distribution of their InferRead DR chest scores compared to those who had Xpert results?

**Response:** We did NOT include any observations with missing Xpert results in our study.

**Comment 5:** You stratified the analysis by age. Did you impute the missing age for the participants who had missing age to mitigate the potential bias? If not, this may need to be highlighted as the limitation in the study.

**Response:** We did not impute the age for participants missing age, however we included them in the analysis, but we were unable to compute sensitivity, specificity, NPV and PPV estimates for these participants. We have highlighted this as a limitation in our study.

**Comment 6:** As the performance of the CAD software varies from region to region, it would be helpful to mention the region/country where the study was conducted.

**Response:** We have revised the manuscript title to include the region where the study was conducted, as you suggested. The updated title now reflects the study location in Lusaka, Zambia.

**Comment 7:** Age, HIV status and history of previous TB are mentioned as the stratifying variables but no definition of the population whose images were analyzed. Which population was considered? What was the inclusion criteria?

**Response:** We have clarified the population and inclusion criteria in the revised manuscript. The images analysed were from individuals who participated in an Active TB Case Finding TB study in Lusaka, Zambia. The inclusion criteria required participants to be adults (≥15 years) identified as having chest X-ray, sputum evaluation and TB diagnosis results based on Xpert or culture tests. We have updated the methods section to define the population more explicitly.

**Comment 8:** Where was the study conducted? i.e From which region/country were the data collected?

This should be included in the methods section.

**Response:** We have updated the methods section to explicitly mention that the study was conducted in Lusaka, Zambia.

**Comment 9:** The authors refers Xpert as the” gold standard”. This gives an impression that Xpert diagnoses TB with certainty. This is not true as Xpert is known to have false positive and false negative rates. Using the term reference standard may help acknowledge the limitations of Xpert. These limitations may need to be highlighted in the discussion as potential limitations to accurate evaluation of InferRead DR Chest.

**Response:** We acknowledge the limitations of Xpert, which can produce both false positives and false negatives. In the revised manuscript, we have replaced the term "gold standard" with "reference standard" to reflect this. Additionally, we have highlighted the limitations of Xpert in the discussion, noting its potential impact on the accurate evaluation of InferRead DR Chest, particularly considering these diagnostic challenges.

**Comment 10:** Line 44: Use of the term” Previous history of TB”. I think” history of previous TB” sounds more appropriate.

**Response:** We have corrected the phrase "Previous history of TB" to "history of previous TB" in Line 44, as well as in all other instances where it appears in the text.

**Comment 11:** In the methods section, estimation of the performance of InferRead DR Chest as a function of age, HIV status and history of previous TB is reported to have been conducted. However, there are no results. Can these be reported?

**Response:** We have now included the AUCs of InferRead DR Chest performance as a function of age, HIV status, and history of previous TB in the results section, as requested.

**Comment 12:** Adding descriptive results of the population might help to quickly understand the sample analyzed upon reading the abstract.

**Response:** We have added descriptive results of the population, including key demographic variables, to the abstract.

**Comment 13:** The authors make a conclusion on the need for further training of InferRead DR chest. This is not supported by the data in the abstract. I would recommend adding results to justify this claim.

**Response:** We have revised the abstract to include relevant results supporting the need for further training of the InferRead DR Chest. This justification is now clearly reflected in the conclusion.

**Comment 14:** Line 106-107: The authors wrote that a score closer to or equal to 100 indicate the likelihood of TB-related abnormality. While higher scores are associated with higher likelihood of TB, this sentence is not correct as the authors established a threshold score of 10 as suggestive of TB. I suggest this sentence be rewritten.

**Response:** We have updated the manuscript to clarify that while higher scores are associated with a greater likelihood of TB-related abnormalities, a threshold score of ≥0.1 was established as suggestive of TB in this study. Please also note that the manuscript has been updated to reference a score between 0 and 1 which were values generated by the CAD system. Initially, we converted these values by multiplying by a 100 for analysis purposes. This latest submission maintains the original values.

**Comment 15:** Line 112: The use of the term ”gold standard” needs attention as mentioned in the general comments and in the abstract above. Even culture, which is known to have a higher accuracy for TB diagnosis has less than optimal sensitivity. Hence, it can’t be referred to as a gold standard as reported by the authors in line 263.

**Response:** We have replaced the term "gold standard" with "reference standard" throughout the manuscript, including in the abstract, to acknowledge the limitations of both Xpert and culture in TB diagnosis.

**Comment 16:** Lines 115-117: As one of the goals of the study was to determine a feasible threshold score for evaluation of InferRead DR chest, shouldn’t this be presented in the results section?

**Response:** We appreciate the reviewer’s suggestion. The description of determining a feasible threshold score for InferRead DR Chest is presented in the methods section because it outlines the process and criteria, we used to establish the threshold. This section aims to provide clarity on how the threshold was derived, ensuring transparency in the study design and analysis. The corresponding sensitivity and specificity values based on this threshold are reported in the results section. We believe this structure provides a clear distinction between the methodology employed and the performance outcomes.

**Comment 17:** Line 127: InferRead Chest DR? or InferRead DR Chest?

**Response:** It is InferRead DR Chest. This has been corrected throughout the manuscript were InferRead Chest DR is being referred to.

**Comment 18:** As mentioned in the general comments above, this section may need to be expanded. For example, give a comparison of InferRead DR Chest to other AI-based methods suchCAD4TBv6 & V7. For my own understanding, the threshold score of 10 derived for this screening tool is close to the values of 10 & 18 for CAD4TB version 7 obtained elsewhere(<https://doi.org/10.1371/journal.pone.0305126>). Are they based on the same Algorithm? Handling of Xpert trace results may need to be mentioned. You mentioned the need for further training of InferRead DR chest to improve accuracy. Given the performance and the possibility for improved accuracy upon further advancement, is this screening tool affordable? Does it meet the other WHO recommended properties for use as a triage test?

**Response:** We have expanded the section to provide a comparison of InferRead DR Chest with other AI-based methods, including CAD4TB, as requested. While the threshold score of 0.1 we derived is close to the thresholds reported for CAD 4 TB, the algorithms are based on different proprietary AI systems. This has been clarified in the revised text.

Additionally, we have addressed the handling of Xpert trace results, stating that they were not included in the reference standard for this study.

Regarding the affordability and WHO criteria, while the study demonstrates promising accuracy, we acknowledge that further advancements in training the algorithm could improve its performance. Affordability and adherence to other WHO-recommended properties, such as cost-effectiveness and feasibility as a triage test, remain critical considerations, and further research is needed to assess these factors fully.

**Reviewer #2 Comments:**

**Comment 1**: The opening paragraphs describe the global challenges of TB detection and the need for additional tools. However, the authors should be careful not to infer that CAD is a TB diagnostic. CAD has been recommended by the WHO for screening/triage testing. CAD cannot diagnose TB alone and therefore microbiological diagnostic testing (e.g., NAAT, culture) is still required. It is suggested the authors ensure this important point is reflected.

**Response 1**: We have revised the manuscript to explicitly state that CAD is a screening and triage tool, as recommended by the WHO, but is not a standalone diagnostic tool for TB. We have emphasized that microbiological diagnostic tests, such as NAAT and culture, are still required for a definitive TB diagnosis. This change has been incorporated in the Introduction section to avoid any potential misinterpretation of CAD’s role.

**Comment 2:** Line 76. Although noted that InferRead DR Chest is from InferVision, it is recommended that it be written ‘InferRead DR Chest (InferVision, China)’.

**Response 2:** We have revised the manuscript to include the recommended clarification. It now reads: "InferRead DR Chest (InferVision, China)" to ensure the proper identification of the source of the technology.

**Comment 3:** The authors note that the primary objective of this manuscript was to assess the overall performance of InferRead DR Chest, including a threshold that optimizes sensitivity. However, the authors do not highlight any previous literature that has evaluated this CAD software. This is important and sets the stage for the reader, assisting them to know what has already been reported and how this study differs and adds to the evidence base.

**Response 3:** We appreciate the reviewer’s comment and have highlighted previous literature that has evaluated the CAD software in question in the discussion section.

**Comment 4:** As a diagnostic accuracy study. The authors are encouraged to present their methodology according to STARD guidelines: (i) Bossuyt PM, Reitsma JB, Bruns DE, Gatsonis CA, Glasziou PP, Irwig L, et al, For the STARD Group. STARD 2015: An Updated List of Essential Items for Reporting Diagnostic Accuracy Studies, (ii) <https://bmjopen.bmj.com/content/6/11/e012799>).

**Response 4:** We have revised the methods section in the manuscript to align with the STARD guidelines for reporting diagnostic accuracy studies.

**Comment 5:** The authors report that radiological images from the TB REACH Wave 5 study were evaluated by InferRead DR Chest. It would be helpful to briefly summarise this study in the current manuscript in addition to citing the study. Furthermore, what did Wave 5 entail? How many were screened in the parent study and how many of those were included in the current study?

**Response 5:** In response, we have briefly summarized the study by Kagujje et al., titled Active TB case finding in a high burden setting; comparison of community and facility-based strategies in Lusaka, Zambia, and incorporated it into the manuscript. We reference this study as an active case finding study and have gotten rid of the TB REACH Wave 5 study reference.

**Comment 6:** Line 103. Further details are required about the CAD analysis. For example, were images sent to InferVision for analysis? Was it done by the authors on a server provided by InferVision? Was there any training involved? To what extent were the developers involved?

**Response 6:** To clarify, the images were sent to InferVision, where they were processed using the InferRead Chest DR algorithm on their server. The authors were not involved in processing the images, and no additional training of the algorithm was required, as it was already pre-trained. The developers' involvement was limited to providing technical support during the image processing phase, ensuring that the system functioned properly. They were not involved in the study design, data analysis, or interpretation of results. This information has been incorporated into the revised manuscript for clarity.

**Comment 7:** The data analysis section could be refined and truncated.

**Response 7:** The analysis section has been refined and truncated.

**Comment 8:** Line 110. The full name, company, and version of the statistical software used should be reported.

**Response 8:** The full name, company, and version of the statistical software used has now been specified in the manuscript.

**Comment 9:** Lines 115-117. As one of the main aims of the study, the identification of the optimal threshold should not be reported in the methods, but rather the results section along with the threshold’s corresponding sensitivity and specificity.

**Response:** We have revised the manuscript to ensure that the identification of the optimal threshold, along with its corresponding sensitivity and specificity, is now reported in the Results section. Additionally, as per your guidance and the STARD guidelines, the test methods have been clearly described in the Methods section, while the detailed findings related to the threshold’s performance are appropriately presented in the Results. We hope this revision addresses the concern.

**Comment 10:** I am not convinced that the analysis for the NNS is relevant or sound in this analysis. Please can the authors clarify their rationale and provide specific details in the data analysis section as it is currently limited on details.

**Response:** The number needed to screen (NNS) was calculated to estimate the number of individuals that would need to be screened in the population of interest to identify one TB patient using the selected threshold. This analysis was particularly relevant for understanding the practical application and efficiency of the CAD system in facility settings, where it could be implemented for active TB case finding.

**Comment 11:** It would be more readable if the authors presented their results in a more systematic manner. For example, report the included participants and exclusions, then demographics, then threshold(s) and accuracy estimates. Lines 161-162. Please could the authors provide more details on the sensitivity analysis. Perhaps add as a supplement?

**Response:** We have reorganized the Results section to present the data in a more systematic manner, starting with the included participants and exclusions, followed by the demographic characteristics, and finally reporting the threshold(s) and accuracy estimates.

Additionally, we have clarified the text referred to as the sensitivity analysis. The wording was corrected, as it was previously an error. We have now provided a supplementary file (**chi-squared test of association results**) that details the investigation related to missing age data, as well as a second supplement file (**sensitivity analysis results**) outlining how the actual sensitivity analysis was conducted. These changes aim to improve clarity and enhance the overall presentation of our findings.

**Comment 12:** To improve readability and understanding, it is suggested that the authors report the total number of patients screened in the parent study and the percentage of those included in the current manuscript. Furthermore, it would be helpful to report the number of TB-positive individuals in the study in the text, as opposed to only in Table 1**.**

**Response:** We have addressed this suggestion by reporting the total number of patients screened in the parent active case finding study, as well as the percentage of those included in the current manuscript. Additionally, the number of TB-positive individuals has been explicitly stated in the text, not just in Table 1, to improve readability and understanding. These revisions have been made to ensure clearer reporting of key study details.

**Comment 13:** Fig 1. The TP, FP, TN, and FN would fit better if reported with the accuracy estimates lower down in the results section.

**Response:** We have moved these details to the results section where the sensitivity, specificity, and other performance metrics are discussed, ensuring a more streamlined presentation.

**Comment 14:** Table 1. The authors report ‘TB Xpert or culture test result’ but only Xpert in the text of the methods section. Can the authors please clarify what the reference standard was. This is critical as the interpretation of CAD accuracy will be different.

**Response:** We have clarified that the gold standard test results were based on either Xpert or culture tests, and this has been explicitly specified in the Methods section of the revised manuscript. A TB-positive diagnosis was defined as being either Xpert-positive or culture-positive. We hope this addresses your concern and ensures clearer interpretation of the CAD accuracy in relation to the reference standard.

**Comment 15**: Table 1. The authors have provided percentages confusingly. It would be easier for the reader if the percentage were out of the TB-negative and TB-positive groups per column (i.e., out of 1601 and out of 289, respectively).

**Response:** We have updated Table 1 to show percentages calculated based on the total number of TB-negative (1601) and TB-positive (289) individuals in each column. This should make the comparison more intuitive and easier for readers to follow.

**Comment 16:** Lines 174-178 and Table 2. Although I can vaguely understand what the authors are trying to report, the way in which the authors present their performance results is rather confusing. One cannot say ‘an overall sensitivity of 94.1%’ as sensitivity will change depending on your threshold. It appears the authors have set the threshold first and then reported the corresponding sensitivity and specificity. In a retrospective analysis such as this, it would make more sense to show the different sensitivities and specificities across multiple thresholds and then determine the optimal threshold (e.g., a threshold that corresponds to 90% sensitivity). Furthermore, why was 10 chosen as the optimal threshold with a sensitivity of 94.1%? Was there a slightly higher threshold that had ~90% sensitivity but improved specificity?

The threshold cannot be ‘>10’ – this is confusing. A CAD threshold is a single number resulting in a binary classification (i.e., positive if above and negative if below).

**Response:** In this study, we selected a single threshold of ≥0.1, which aligns with the WHO guideline for TB screening tools. According to the guideline, a screening test should have a minimum sensitivity of 90% and a specificity of 70% to detect or rule out pulmonary TB in individuals being screened (WHO Consolidated Guidelines on Tuberculosis - Module 2: Screening - Systematic Screening for Tuberculosis Disease, 2021). We created a binary variable for the CAD results, assigning a value of 1 if the TB score was ≥0.1 and 0 if the TB score was <0.1 after reviewing the different sensitivity estimates produced at different tb score thresholds. At this threshold InferRead DR Chest achieved a sensitivity of 94.1% and a specificity of 26.9%.

We were particularly focused on achieving high sensitivity, as the CAD tool is intended to be used as a screening tool rather than a diagnostic one. In this context, it is critical that the tool accurately identifies potential TB cases for further diagnostic testing. Additionally, we have provided a supplementary file (roctab detail results & roctab detail results continued) that includes select sensitivity estimates at various TB scores to give you a sense of the tool's performance.

**Comment 17:** Lines 181-189 and Fig 2. It is clinically relevant and strengthens the study to have sub-group analyses. The authors show AUCs were lower for males, older individuals, those with a history of previous TB, and people living with HIV. These findings corroborate findings of previous studies. However, did the authors compare AUCs statistically? Were there significant differences?

**Response:** We agree that subgroup analyses are clinically relevant and strengthen the study. In response to your query, we compared the area under the curve (AUC) statistically to assess whether there were significant differences. The statistical comparisons showed significant difference only for individuals with a history of previous TB and have incorporated the statistical tests results in the revised manuscript.

**Comment 18:** Line 204-205. The authors may want to do an updated literature search as there are several publications that have evaluated InferRead for TB screening. For example, (i) Codlin AJ, et al. Independent evaluation of 12 artificial intelligence solutions for the detection of tuberculosis. Sci Rep. 2021;11(1):23895; (ii) Qin ZZ, et al. Tuberculosis detection from chest x-rays for triaging in a high tuberculosis-burden setting: an evaluation of five artificial intelligence algorithms. Lancet Digit Health. 2021;3(9):e543-e554; (iii) Qin ZZ, et al. Computer-aided detection of tuberculosis from chest radiographs in a tuberculosis prevalence survey in South Africa: external validation and modelled impacts of commercially available artificial intelligence software. Lancet Digit Health. 2024:S2589-7500(24)00118-3.

**Response:** We have conducted an updated literature search and clarified in the text the one study done in Africa we were referring to.

**Comment 19:** Lines 212-217. The authors may want to reword their point on CAD ‘demonstrating good sensitivity’. Please remember that you have retrospectively set the threshold to report that sensitivity (with a corresponding low specificity of ~26.9%). If you changed the threshold, your sensitivity and specificity would also change.

**Response:** We acknowledge that the threshold for the CAD score was set retrospectively to achieve the reported sensitivity, and that adjusting the threshold would result in changes to both sensitivity and specificity. Considering this, we have reworded the relevant section of the manuscript to clarify this point and align with your guidance.

**Comment 20:** It would be beneficial for the authors to expand on the clinical implications of the findings. For example, having a low specificity (therefore, many false positives) will result in increased costs for additional diagnostic microbiological testing.

**Response:** We agree that expanding on the clinical implications of our findings is important, particularly concerning the low specificity of the CAD tool and its impact on additional diagnostic costs. We have addressed this point in the revised manuscript and provided a discussion on the potential consequences of a high false-positive rate in clinical settings.

**Comment 21:** It is suggested that the authors compare their findings to similar studies and discuss similarities and differences in terms of methods, results, etc.

**Response:** We have discussed the performance of InferRead DR Chest in relation to other AI-based CAD systems, such as CAD4TB and qXR.

**Comment 22:** Lines 242-247. The authors should re-evaluate their strengths and limitations section. For strengths, the authors report a ‘substantial sample size’ yet have not included sufficient detail about the population studied (e.g., active vs passive case-finding). The authors may want to include having a microbiological reference standard as a strength. For limitations, the authors may need to consider noting that Xpert MTB/RIF was used and not the more sensitive Ultra which may influence accuracy estimates of CAD. Furthermore, the authors may want to add that they may not be able to comment on CAD accuracy to detect sub-clinical TB (as >90% of participants were symptomatic).

**Response:** We acknowledge the need to enhance the strengths and limitations section to provide more detailed insights into the population studied and the diagnostic tools used. We have clarified the nature of the population and noted the limitations regarding the use of Xpert MTB/RIF.

**Comment 23:** Although one can work out what the authors conclude, it is suggested that the authors focus on aligning the primary aim and their findings in the conclusion (e.g., InferRead DR Chest performance did not meet expectations / it did not meet WHO TPP / at the optimal threshold (sensitivity 90%) specificity was low, etc.).

The authors bring in new ideas into the conclusion which may be better suited to the discussion section.

**Response**: We have revised the conclusion.

**Comment 24:** The abstract could be more concisely written, especially the methods section. This could be truncated to allow more findings to be reported in the results section. The authors may want to amend the abstract after reviewing the comments above.

**Response:** We revised the abstract for it to focus on the most critical aspects and allow more space for reporting the key findings in the results section.

Thank you for your time and consideration. We look forward to your feedback.

Sincerely,

**Paul Somwe**

Centre for Infectious Disease Research in Zambia (CIDRZ)

Email: [paul.somwe@cidrz.org](mailto:paul.somwe@cidrz.org)

Mobile number: +260966840869
