# Supplementary material for: Evaluating InferVision’s Computer-Aided Detection (CAD) algorithm for Tuberculosis (TB) screening, Lusaka, Zambia
Source: PLOS Glob Public Health. 2025 Jun 18;5(6):e0003955. doi: 10.1371/journal.pgph.0003955 (PMC12176154; doi:10.1371/journal.pgph.0003955)
Supplement: Attachment — Submitted filename: Response to Reviewers..docx [file pgph.0003955.s006.docx]

**31^st^ March 2025**

**Emily B. Wong**

Academic Editor

PLOS Global Public Health

Dear Emily,

Thank you for sharing the second round of reviewer comments on our manuscript titled ***“Evaluating the Performance of InferVision’s Computer-Aided Detection (CAD) Algorithm for Tuberculosis (TB) Screening, Lusaka Zambia.”*** We appreciate the valuable feedback provided by you and the reviewers, and we remain committed to thoroughly addressing each point.

We have addressed each of the reviewer’s comments below, providing detailed responses and revisions as appropriate. All changes have been highlighted as tracked changes in the revised manuscript to clearly illustrate the modifications based on this feedback.

**Reviewer #1 Comments:**

**Comment 1:** I am still confused as to why the threshold is being stated in the methods section. Part of the aim of this study was to evaluate for an optimal threshold that matched a sensitivity of 90%. The authors are therefore stating results in the methods section. Can the authors clearly explain why they chose to report this finding/result in the methods section.

**Response:** We have addressed this by moving the reference to the threshold from the methods section to the results section, as guided. This ensures that the identification of the threshold, as a study finding, is now appropriately presented alongside the corresponding performance metrics.

**Comment 2:** Although the authors respond to the original comment, can the authors please clearly explain why they still use the ‘greater than or equal (≥)’ symbol for the threshold, as it is still confusing to me. A threshold is a single number between 0 and 1 (or 0 and 100) corresponding to a certain sensitivity and specificity. It cannot be above or below that number as the respective sensitivity and specificity would change. Currently the authors state that “At 94.1% sensitivity, InferRead DR Chest had a specificity of 26.9% at a TB score threshold of ≥0.1”. It cannot be greater than or equal to 0.1, it is 0.1. Anything above (or below) 0.1 would have a different sensitivity and specificity.

**Response:** To avoid confusion around the concept of a fixed diagnostic threshold, we have revised the wording in the manuscript. Instead of referring to "a threshold of ≥10," we now state that “At a TB score cut point of 0.12, which we determined to align with the WHO’s minimum sensitivity requirement of 90% for TB screening tools, the InferRead DR Chest showed an overall sensitivity of 90.3% (95% CI: 86.3%—93.5%) and an overall specificity of 39.2% (95% CI: 36.8%—41.7%).”

**Comment 3:** Please can the authors note how they managed Xpert trace results. I did not find mention of it in the manuscript.

**Response:** We confirm that only observations with confirmed MTB-positive or MTB-negative results were included in the analysis, and any observations with Xpert trace results were excluded (This is stated in line 110 of the revised manuscript with tracked changes). This approach was taken to ensure a clear binary composite reference standard.

**Comment 4:** I appreciate the authors adding a brief description of the parent study. However, please can the authors mention why only ~2,000 X-rays of the 18,000 individuals screened were included in the current study. Were these selected? If so, how? Were these the only images available?

**Response:** Thank you. These x-rays were not just selected. Among the ~18,000 individuals, only 10,763 had CXR and of these only 2212 had submitted sputum and had complete reference test results and a CXR result.

**Comment 5:** My previous comment on whether there were slightly higher thresholds that had ~90% sensitivity but improved specificity was not completely addressed. Although I received the supplementary material, these were image files and only had thresholds ranging from 0.0479 to 0.102305. Where are the other thresholds? For example, what were the sensitivities and specificities of thresholds between 0.1 and 0.2?

**Response:** Thank you for your follow-up comment. We have now shared an Excel file named **roctab_detail_results** that includes a full range of thresholds, including those between 0.1 and 0.2, along with their corresponding sensitivity and specificity values.

**Comment 6:** I would like to see a more structured supplementary material. I do not believe several image files is sufficient.

**Response:** A more structured supplementary material has now been provided. The Excel file named “**roctab_detail_results”** contains a full range of threshold values with corresponding sensitivity and specificity estimates. Additionally, the two STATA outputs previously shared as image files are now clearly presented in a Word document named **"Supplementary file,"** including a summary of what each output represents and explanations to aid in interpretation. We hope this revised format improves clarity and addresses your concerns.

**Reviewer #2 Comments:**

**Comment 1**: Lines 38 & 56: The reported age needs to include units (years)

**Response 1**: Thank you for bringing this to our attention. We have revised the text to include the appropriate unit of measurement, specifying age in years.

**Comment 2:** The authors specified the” WHO target product profile” as” WHO target

profile”. This should be specified correctly.

**Response 2:** We have corrected the wording to "WHO target product profile" to accurately reflect the proper terminology.

**Comment 3:** Line 61:” tb” should be capitalized i.e.” TB” as it is an acronym

**Response 3:** We have corrected the text by capitalizing “TB” to reflect its proper usage as an acronym.

**Comment 4:** Lines 94 & 95: References 19 & 21-22 are disjoint.

**Response 4:** We have reviewed and corrected the references to ensure they are properly connected and accurately cited.

**Comment 5:** The cited reference for the WHO TPPs is not correct; Consider the reference in this link <https://iris.who.int/bitstream/handle/10665/135617/WHO_HTM_TB_2014.18_eng.pdf>

**Response 5:** Thank you for your comment. We have made the necessary changes to ensure that the correct, originally referenced WHO document is cited. The citation now accurately reflects the source outlining the target product profile for TB screening tools.

**Comment 6:** Line 142: Might be helpful to add the facility and community-based approaches?

**Response 6:** Thank you, we have added some detail of the case finding activities in lines 123-127 in the revised manuscript with tracked changes.

**Comment 7:** Line 149-150; The statement” ... a threshold score of ≥0.1 was established as suggestive of TB in this study.” is a finding and should be reserved to the Results section.

**Response 7:** We have addressed this by moving the reference to the threshold from the methods section to the results section, as guided. This ensures that the identification of the threshold, as a study finding, is now appropriately presented alongside the corresponding performance metrics.

**Comment 8:** Was composite reference standard derived? If so, it needs to be spelt out clearly. See Table 1.

**Response 8:** Thank you for your comment. This study used a composite reference standard, which has now been clearly indicated in the revised manuscript.

**Comment 9:** Line 155: This sentence mentions the findings” ... was a score of ≥0.1”. Delete this to make the sentence grammatically correct. Findings should go to the Results section.

**Response 9:** We have addressed this by moving the reference to the threshold from the methods section to the results section, as guided. This ensures that the identification of the threshold, as a study finding, is now appropriately presented alongside the corresponding performance metrics.

**Comment 10:** Line 155-158: Consider revising the reference to the correct source in <https://iris.who.int/bitstream/handle/10665/135617/WHO_HTM_TB_2014.18_eng.pdf>

**Response 10:** Thank you for suggesting revising the reference to the correct source. Upon review, we have updated the citation to the appropriate WHO document: "High-priority target product profiles for new tuberculosis diagnostics: report of a consensus meeting, 28-29 April 2014, Geneva, Switzerland."

**Comment 11:** Line 163-164: The statement” ... with a score of ≥0.1 identified as meeting the WHO recommended sensitivity of 90%” is a finding and should be reserved to the Results section.

**Response 11:** Thank you for your comment. Again, we acknowledge that the statement refers to a finding. We have addressed this by moving the reference to the threshold from the methods section to the results section, as guided. This ensures that identifying the threshold, as a study finding, is now appropriately presented alongside the corresponding performance metrics.

**Comment 12:** How was the reference standard defined? If a composite of Xpert MTB/RIF and Culture then that should be mentioned explicitly in this subsection. Table 1 seems to suggest that a composite reference standard was derived.

**Response 12:** We confirm that the reference standard used in this study was a composite of Xpert MTB/RIF and culture test results. This has now been explicitly stated in the relevant subsections of the manuscript to ensure clarity and alignment with the data presented in Table 1.

**Comment 13:** Lines 175-177: The justification for the calculating the NNS should be reserved to the discussion. Please move it.

**Response 13:** The intention of the NNS statement in the Methods section was not to justify its relevance but rather to describe the analysis conducted as part of the study. It was included to outline the procedures involved in NNS calculation. However, we have revised the wording to ensure it aligns as a procedural description rather than a justification, reserving all interpretive or explanatory content for the Discussion section**.**

**Comments 14, 15, & 16:** Line 240-241: This sentence seems to suggest that the threshold was set to allow evaluation of the index test. This is not true as the threshold was determined based on the WHO recommended minimum requirements on the sensitivity of the targeted product. Add a sentence before this sentence to reflect it as such. The sentence included in the abstract is well written.

- Line 254:” Based on the set threshold of ≥ 10,” This threshold was not set but

determined based on WHO minimum requirement of 90% sensitivity set by WHO

for targeted products.

Lines 254-256: Revise this sentence as mentioned in the second bullet above.

**Response 14, 15, & 16:** Thank you for your feedback. The comment has been addressed by clarifying that the TB score threshold of ≥0.1 was determined based on the World Health Organization's (WHO) minimum sensitivity requirement for TB screening tools, rather than being arbitrarily set. The revised text now accurately reflects this.

**Comment 17:** Lines 267:” ... and individuals with history of previous TB showed” needs to be revised to read”... and individuals with a previous TB history of previous TB yielded”

**Response 17:** All sentences that read “previous history of TB” have now been revised to read “history of previous TB”

**Comment 18:** Table 1 & Table 2: Revise” Previous history of TB” to” History of previous TB”

**Response 18:** Text has been revised to read History of previous TB.

**Comment 19:** HIV status results -” Indeterminant” or” Indeterminate”. I think the later.

**Response 19:** Text has been updated to indeterminate.

**Comment 20:** Lines 313-314: This interpretation is incorrect and even contradicts the interpretation in Lines 337-340. The specificity of InferRead DR Chest were similar for males both females. While the female gender had a higher specificity, the 95% confidence intervals of specificity for both males & females are overlapping. Hence, the difference is not statistically significant.

**Response 20:** Upon closer examination of Table 2, you will notice that the confidence intervals for specificity estimates for males and females **do not overlap**, indicating a statistically significant difference in specificity between the two groups. However, the confidence intervals for the AUC estimates for both males and females **do overlap**, suggesting that the overall diagnostic performance of InferRead DR Chest, as measured by AUC, is not significantly different between the sexes.

Thank you again for your support throughout this review process.

Sincerely,

**Paul Somwe**

Centre for Infectious Disease Research in Zambia (CIDRZ)

Email: [paul.somwe@cidrz.org](mailto:paul.somwe@cidrz.org)

Mobile number: +260966840869
